# A Peculiarity in the Course of the Radial Artery

**Published:** 1801-03

**Authors:** John Yelloly

**Affiliations:** Bartlett's Buildings


### *A Peculiarity in the Course of the Radial Artery, communicated to the Editors by* Dr. Yelloly.

THE divifion of the Brachial Artery into two branches near the fhoulder, or in the middle of the arms, is a circumftance noticed by moft authors who write on the blood-veffels, and does not unfrequently occur. I have obferved, however, a peculiarity in the courfe of the radial artery, which it may not perhaps be improper to notice, as it is not mentioned by Sabatier, Mr. J. Bell, or Prof. Murray, though known to a few gentlemen with whom I have converfed. It is this, that the artery, inftead of directing its courfe as in the ufual way, on the infide of the radius, through the whole length of it, fometimes fuddenly turns outwards, about an inch above the place at which the pulfe is ufually felt, goes over the bone, and continuing its courfe along the top of, or near the outfide of it, can be diftinctly felt going deep into the fpace between the metacarpal bones of the thumb and fore finger. In fuch cafes, a fmall branch only is found in the ufual direction of the trunk; and this branch can be traced going towards the palm. It feems to anfwer to that which is fent off about the place at which the radial artery ufually makes its bend outwards, under the tendons of the extenfors of the thumb. I firft had occafion to obferve this lufus naturæ, about three years ago, in a robuft man, with a recent attack of fever. The pulfe was extremely fmall, very different from what his general appearance would have led me to expect. That of the other arm was very ftrong and full. On examining more minutely into the caufe of this difference, it was found to be what is above ftated. Since that period, I have feen the fame occurrence in about eight inftances, one of them in a female, and two in the fame perfon. Phænomena of this kind afford in general little practical inftruction. Had I not been aware however of this deviation, I might perhaps, in fome of the inftances, have been induced to have drawn a more unfavourable conclufion on the nature of the difeafe, than the circumftances warranted. I am, &c.

